# Deterioration in Clinical Status Is Not Enough to Suspend Eculizumab: A Genetic Complement-Mediated Atypical Hemolytic Uremic Syndrome Case Report

**DOI:** 10.1155/2019/9264824

**Published:** 2019-07-09

**Authors:** Luca Calvaruso, Alessandro Naticchia, Pietro Manuel Ferraro, Gisella Vischini, Stefano Costanzi

**Affiliations:** ^1^U.O.C. Nefrologia, Fondazione Policlinico Universitario A. Gemelli IRCCS, Rome, Italy; ^2^Università Cattolica del Sacro Cuore, Rome, Italy

## Abstract

**Background:**

Atypical hemolytic uremic syndrome (aHUS) is characterized by microangiopathic hemolytic anemia, thrombocytopenia, and renal failure. Mutations in CFI gene coding for complement regulation factors and in THBD gene coding for endothelial cell receptor thrombomodulin could predispose to the disease and hypertension can trigger the onset.

**Case Presentation:**

A 51-year-old female patient who had received kidney transplant eighteen years ago presented with hypertensive peak and hemolysis pattern. Normal ADAMTS13 levels as well as negative culture and serology for Shiga-toxin excluded, respectively, thrombotic thrombocytopenic purpura (TTP) and typical HUS caused by Shiga toxin-producing* Escherichia coli* (STEC-HUS). In suspicion of aHUS, we administered eculizumab and hemodialysis sessions were started as the patient showed severe renal failure. After an initial response, the patient developed cerebral hemorrhage. After last eculizumab administration, according to hematological parameters, an unsatisfactory response was observed: given the worsening clinical scenario, we withdrew eculizumab. Pathogenic mutations in CFI and THBD genes were found. After eculizumab reinitiation, looking at hemolysis indexes, we observed a suboptimal response as well as an otherwise adequate renal one: renal graft function was recovered despite persistence of hemolysis signs, after 6 months on regular dialysis.

**Conclusion:**

For the first time, we report an aHUS case in which a peculiar combination of mutations in CFI and THBD is found. We describe the importance of continuing eculizumab despite deterioration of patient's clinical conditions.

## 1. Background

Thrombotic microangiopathy (TMA) is characterized by a clinical presentation with thrombocytopenia, microangiopathic hemolytic anemia (MAHA), and organ injury (kidney, CNS, gut, etc.) [1].

Atypical hemolytic-uremic syndrome (aHUS) belonging to the group of TMA is a genetic or acquired ultrarare, life-threatening disease mainly associated with complement dysregulation. aHUS has an incidence of approximately 0.5 per million per year [2].

Mostly, we refer to aHUS as a hereditary complement-mediated disease [3]. The underlying genetic mutations which predispose to aHUS have incomplete penetrance so that a second hit is required for disease manifestation [4].

On the other hand, pathogenic variants responsible for permanent complement dysregulation are detected in about 40 to 60% of patients with aHUS [5].

Since diagnosis can often be challenging, we can adopt a predictive model for the initial diagnosis of TMA based on LDH and platelet count which was found to accurately predict the presence of TMA [6].

In the pre-eculizumab era, aHUS was characterized by significant morbidity and mortality, with 25% of patients dying, 50% progressing toward end-stage renal disease, and 40-80% experiencing disease recurrence after transplantation [7].

To date, four prospective clinical studies conducted in patients with aHUS have shown efficacy of eculizumab, a monoclonal antibody that specifically binds to the terminal complement protein C5, in aHUS treatment [2, 8].

## 2. Case Presentation

Here, we report a 51-year-old female patient who was referred to our hospital due to hypertensive peak and acute kidney injury (AKI) on a background of chronic kidney disease.

The patient whose primary cause of CKD remains unknown reached ESRD at age of 19. In 1988, she received a cadaveric renal transplantation. The posttransplant therapy included cyclosporine and prednisone. After eighteen years, serum creatinine increased from 132.63 to 707.36 *μ*mol/L [normal laboratory values 44-88 *μ*mol/L]. A kidney biopsy was performed in suspicion of acute rejection and graft failure. The biopsy did not show neither features of cellular rejection nor features of antibody mediated rejection. Furthermore, a suspicion for polyomavirus nephropathy was ruled out as no significant polyomavirus DNA replication was detected on quantitative PCR. No significant lesions related to CNI toxicity such as nodular hyalinosis and striped fibrosis nor isometric vacuolization of tubules were found.

Cyclosporine therapy was suspended, starting therefore immunosuppressive therapy with tacrolimus and prednisone. AKI gradually regressed. Last creatinine baseline value was 158.4 *μ*mol/L.

After three weeks the patient was referred from emergency room to our hospital (February 2^nd^, 2018) due to hypertensive peak and pulmonary edema.

Basic laboratory investigations showed a pattern of hemolysis: LDH 1,014 IU/L [normal laboratory values <250 IU/L], bilirubin 39.33 *μ*mol/L [normal laboratory values 5.13-20.52 *μ*mol/L], platelets 121,000 per microliter [normal laboratory values 150-450 per microliter], severe anemia (Hb 63 g/L) [normal laboratory values 120–150 g/L], and positive finding of schistocytes on the blood smear. ADAMTS13 activity level was normal (51.9%) [normal laboratory values > 50%] and the Coombs test was negative. No neurological signs were present. Moreover, on the first week of February, the patients showed at baseline CKD stage IV with serum creatinine 290.4 *μ*mol/L, eGFR 15.5 ml/min/1.73m^2^, and 24-hour proteinuria 0.84 g.

In suspicion of drug-related TMA, tacrolimus was suspended (tacrolimus blood level was 3.9 ng/ml [normal laboratory values 5–15 ng/mL]) and the dose of mycophenolate mofetil and prednisone was increased. However, no response was observed. The severe anemia (Hb 64 g/L) made blood transfusions necessary.

Given the laboratory and clinical pattern consistent with aHUS, and after having ruled out HUS and secondary causes of TMA, we administered eculizumab 900 mg e.v. after the patient had received meningococcal vaccine and antibiotic prophylaxis using the schedule of 900 mg/weekly for four weeks then 1200 mg starting from the fourth week. Due to worsening AKI with serum creatinine and urea values at 608.33 *μ*mol/L and 21478.5 *μ*mol/L, respectively [urea normal laboratory values 1665.00–3829.50 *μ*mol/L], the patient underwent hemodialysis sessions after positioning a central venous catheter in the femoral vein.

**Figure 1:**
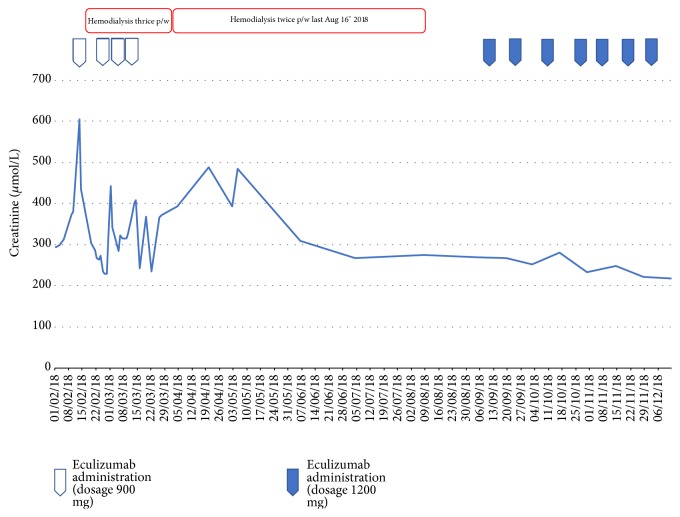
Parameters of serum creatinine across study timeline.

According to hematological parameters, especially platelet count, a quick response to eculizumab was observed two days after administration (Figure 1).

Platelet count increased up to 217,000 per microliter, Hb remained stable in the normal range, and haptoglobin levels and clinical conditions improved.

A second administration of eculizumab was given seven days later. We started observing a poor response to therapy: LDH levels remained out of normal range (686 IU/L) as schistocytes were still present on blood smear.

Due to fever onset, blood cultures were performed and resulted negative, while urine culture showed 366 JC Polyomavirus genome copies per mL whose value gradually decreased to normal range over time of hospital stay. Immunosuppressive therapy with mycophenolate was tapered to 500 mg/die. Research for BK Polyomavirus genome turned out to be negative both in urine and in blood.

Three days later, the patient showed tonic-clonic seizures. On the CT scan, a parietal cortical-subcortical hemorrhage was found. The patient was then transferred to intensive care of our hospital where her clinical picture gradually improved.

During stay at intensive care, atrial fibrillation onset was observed and enoxaparin 2000 UI twice a day was started. Eculizumab was administered twice more with a one-week interval. After the last administration, we observed an unsatisfactory hematological response (low levels of haptoglobin, high levels of LDH, and reticulocyte count) and at the same time oligoanuria, tendency to dyspnea, and ponderal increase so that dialysis sessions were repeated regularly every other day. Given the worsening clinical picture, we suspended eculizumab, despite eculizumab induction therapy being incomplete and CH 50 level being low (<15 Ueq/ml [normal value < 70 Ueq/ml]).

As clinical conditions were gradually getting better, the patient was discharged from our hospital.

**Figure 2:**
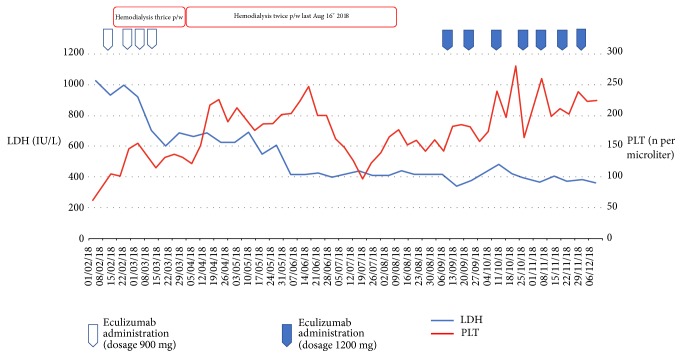
LDH and platelet count across study timeline.

On follow-up, we observed a partial resolution of the hemolysis pattern: no schistocyte was found, LDH levels showed a decreasing trend, and anemia and thrombocytopenia were no longer observed. (Figure 2)

The patient kept on being dialysis-dependent until September.

Genomic analysis of the patient's CFH, MCP/CD46, CFI, C3, CFB, THBD, DGKE, CFHR1, CFHR3, and CFRH5 genes by next generation sequencing (NGS) revealed a pathogenic heterozygous mutation in CFI gene (c.292A>G (p.Thr98Ala)) as well as a heterozygous variant in THBD gene (c.1502C>T (p.Pro501Leu)). The clinical diagnosis of aHUS was confirmed.

On September, in consideration of the mutations found and in order to prevent aHUS relapse, we restarted eculizumab therapy with a fixed dose of 1200 mg every two weeks. After the first administration, we observed a clear improvement of renal function and diuresis as the patient stopped being dialysis-dependent. Nevertheless, according to hematological parameters, the response to therapy had never been complete. Haptoglobin levels, indeed, never rose to normal range after eculizumab administration.

## 3. Discussion

Thrombotic microangiopathies (TMA) are a group of disorders characterized by intravascular microangiopathic hemolytic anemia, thrombocytopenia, and vascular thrombosis. [9]

The currently accepted classification by George et al. describes primary TMAs (known as either acquired or inherited), secondary TMAs, and infection-associated TMAs.

aHUS with complement gene mutation could belong either to hereditary subgroup or to acquired subgroup as, for instance, in the case of aHUS mediated by antibody anticomplement factor H [1].

Several lines of evidence indicate that genetic mutations in the alternative pathway are found in up to half of patients with a clinical diagnosis of aHUS [10].

Finding more than one mutation related to aHUS is rare as around 3% of hereditary complement-mediated aHUS patients have more than one mutation, with increased penetrance per additional mutation [3].

In our case, the patient presented mutations in CFI and THBD genes. As many authors previously reported, CFI mutations do not directly cause TMA, but rather predispose to it [11].

CFI is a factor involved in the downregulation of the alternative pathway. CFI, cooperating with other factors such as CFH and MCP, accelerates the decay of convertase C3bBb, a cornerstone in membrane attack complex activation [12].

As Bresin et al. suggested, CFI seems to have a low pathogenic potential since a mutation of CFI requires another genetic abnormality to induce aHUS. Indeed, several CFI mutations were only found combined with mutations in other genes [13].

Thrombomodulin (TM) regulates coagulation, innate immunity, and complement activation. TM is a transmembrane-type 1-glycoprotein expressed on the vascular endothelium which suppresses clot formation by accelerating thrombin-mediated activation of protein C. Otherwise, thrombomodulin inactivates complement-derived anaphylatoxins C3a and C5a enhancing thrombin-mediated activation of plasma procarboxypeptidase B (thrombin activatable fibrinolysis inhibitor [TAFI]), an inhibitor of fibrinolysis. Mutations in the gene coding for thrombomodulin were previously revealed to be associated with diminished capacity to inactivate C3b [14, 15].

Delvaeye et al. found mutations that jeopardize thrombomodulin function in about 5% of patients with aHUS [15].

Many authors hypothesize that variants of the gene encoding thrombomodulin (THBD) confer a predisposition to endothelial injury that could manifest beside aHUS [14].

In our case, the clinical onset was characterized by hypertensive peak which we can speculate had possibly played the role of TMA trigger. Furthermore, a mutation in THBD was found (c.1502C>T (p.Pro501Leu)).

Van Laeche et al. reported a case of a 33-year-old woman who presented with malignant hypertension and aHUS: the same mutation on the gene coding for TM (C.1502C>T) we noticed was found [16].

The patient showed tonic-clonic seizures and on CT imaging parietal cortical-subcortical hemorrhage. Neurologic involvement is frequently reported. Recently, Schaefer et al. in an analysis of patients with aHUS from the Global aHUS Registry found neurologic involvement in 24% of pediatric and 25% of adult patients, compared with 16% and 8% of children and adults with aHUS previously reported [5, 17].

Eculizumab, a recombinant IgG2/4*κ* humanized monoclonal antibody, was shown to be effective and safe in aHUS treatment. It blocks the cleavage of C5, hindering formation of the proinflammatory peptide C5a and the cytotoxic membrane attack complex C5b-9 [18–20].

The patient discontinued eculizumab therapy due to lack of response and evolving towards end-stage renal disease. Although the main rationale for discontinuing eculizumab therapy is to protect patients from the risk of side effects of meningococcal infection [21, 22] as well as to have a smaller impact on patient's quality of life and on health costs, we took into account the patient's clinical conditions which have been worsening over time. This raises the question if eculizumab should be administered anyway despite the clinical scenario.

As Olson et al. recently stated in a review, several lines of evidence corroborate that risk of relapse after eculizumab discontinuation is high (30%) with unpredictable timing [9].

Some authors observed a relationship between type of complement mutation and the subsequent risk of TMA manifestation [23].

It has also previously been demonstrated that patients with mutations in CFH or THBD genes had the earliest onset of aHUS and the highest mortality [23].

It is worth emphasizing though that, in our case, initial eculizumab induction was not complete: this could explain the above-mentioned suboptimal response as well.

We observed a peculiar clinical presentation as the patient's response to therapy shifted from optimal to unsatisfactory in between the first and the last eculizumab administrations. As the genetic diagnosis was revealed, we restarted eculizumab and obtained an almost complete renal remission. The patient became dialysis-independent. Only serum haptoglobin levels remained low until last observation.

Although the clinical scenario could affect the compliance to eculizumab therapy as well as the risk for meningococcal infection, the response we observed after restart of the drug seems to advocate that eculizumab should not be suspended despite a deterioration of clinical conditions.

In order to provide reliable risk stratification tools and individualize treatment, the development of biomarkers to monitor eculizumab response is mandatory in the future.

To the best of our knowledge, this is the first genetic complement-mediated aHUS case in which such combined mutation in CFI and THBD genes is found and is associated with a suboptimal response to eculizumab.

## Abbreviations

ADAMTS13: A disintegrin and metalloproteinase with a thrombospondin type 1 motif, member 13 (also known as von Willebrand factor-cleaving protease)
aHUS: Atypical hemolytic uremic syndrome
AKI: Acute kidney injury
CFH: Complement factor H
CFI: Complement factor I
MAHA: Microangiopathic hemolytic anemia
NGS: Next generation sequencing
STEC-HUS: Typical HUS caused by Shiga toxin-producing* Escherichia coli*
THBD: Thrombomodulin
TMA: Thrombotic microangiopathy
TTP: Thrombotic thrombocytopenic purpura.
